# Changes of Heavy Metals in Pollutant Release and Transfer Registers (PRTRs) in Korea

**DOI:** 10.3390/ijerph110302381

**Published:** 2014-02-26

**Authors:** Yong-Su Kwon, Mi-Jung Bae, Young-Seuk Park

**Affiliations:** 1 Department of Biology, Kyung Hee University, Seoul 130-701, Korea; E-Mails: davy3021@hanmail.net (Y.-S.K.); mjbae@khu.ac.kr (M.-J.B.); 2 Department of Life and Nanopharmaceutical Sciences, Kyung Hee University, Seoul 130-701, Korea

**Keywords:** heavy metals, Pollutant Release and Transfer Registers, self-organizing map, Toxics Release Inventory (TRI), spatial and temporal changes

## Abstract

Industrial effluent containing heavy metals discharged into streams may pose high toxicity risks to aquatic organisms and to human health. Therefore, it is important to understand how to change the amount of effluent with heavy metals discharged from industries into open aquatic ecosystems both for effective management of heavy metals and to foster sustainable ecosystems. This study was conducted to characterize the release of heavy metals from industries based on the Pollutant Release and Transfer Registers database in Korea from 1999 to 2010. From the database, we selected nine heavy metals (Pb, Cd, Mn, Sb, Cu, Zn, Cr, Sn, and Ni) and compared the differences in their effluent for different types of industries. The heavy metal effluents released into freshwater ecosystems were classified into four clusters through the learning process of the self-organizing map. Cluster 1 was characterized by the relatively higher effluent volumes of heavy metals, whereas cluster 4 had lower effluent volumes. The different patterns of the effluent volumes in heavy metals were closely associated with the differences of industrial types, and the changes of effluents of heavy metals reflected the changes in regulations and laws for aquatic ecosystem management.

## 1. Introduction

Heavy metal contamination is a worldwide environmental problem in the aquatic ecosystems. Mining and industrial processing are the main sources of heavy metal contamination [1,2]. Among these anthropogenic activities, urbanization has caused the detrimental environmental disruption of freshwater ecosystems, especially due to effluents from industrial complexes [3,4,5]. Aquatic organisms have suffered from the inflow of heavy metals in industrial effluents and this could eventually influence human health [6,7,8,9]. Heavy metals may be accumulated to a toxic concentration level that can lead to ecological damage [10]. For instance, fish development is affected by the heavy metals in the early life stages, such as hatching, larval development and juvenile growth [11]. Therefore, a lot of efforts such as strengthening the environmental standards and policies for monitoring aquatic ecosystems have been made worldwide to maintain the water quality as well as aquatic health. 

The US Congress created the Toxics Release Inventory (TRI) pursuant to the Emergency Planning and Community Right to Know Act (EPCRA) after the severe impact from a methyl isocyanate leak by accident in Bhopal, India, in 1984 [12]. The purpose of the TRI system is to provide the public the information on the presence and release of toxic chemicals. The TRI requires manufacturing facilities that use threshold amounts of over 300 chemicals to publically report their estimated annual toxic emissions to land, air, and water, as well as shipments of waste offsite [13]. This environmental policy underlying premise of public disclosure can enable effective and informed participation by various constituencies to exert pressure on manufacturing facilities to improve their environmental performance [14]. Implementation of TRI in USA has resulted in a substantial reduction in emissions to the environment from industrial facilities [14]. As a result, the Organisation for Economic Co-operation and Development (OECD) advised member countries to implement regulations similar to TRI (for instance, National Pollutant Release Inventory (NPRI) in Canada, Pollution Inventory (PI) in England, and National Pollutant Inventory (NPI) in Australia) [15], resulting in the development of Pollutant Release and Transfer Registers (PRTR): PRTR is defined as a catalogue or register of releases and transfers of potentially harmful substances to the environment from a variety of sources [16].

The Ministry of Environment in Korea enforced a PRTR in 1999 based on the recommendation of OECD [17], and developed a standard method for estimating amounts of potentially harmful substances released and transferred prior to the formal implementation of TRI on the national scale [16,18]. The PRTR in Korea is serviced through the internet (http://ncis.nier.go.kr/tri/) to promote voluntary improvements on the management of chemical substances by business operators. It delivers information about hazardous chemical releases at different space and time to business operators and citizens and supports decision informed by all levels of industry, government, non-governmental organization and the public (http://ncis.nier.go.kr/tri/). These PRTR data also provide important information regarding point-source emissions, although they do not contain geographical characteristics [19].

For effective decision making in the management of ecosystem health, it is necessary to understand how the amounts of effluent in heavy metals differ in space and time. Although there are many studies on the changes in water quality and toxicities of heavy metals [20,21,22], there are few comprehensive studies on the changing trends in heavy metals released from industrial areas to public waters on a nationwide scale. Therefore, in this study we aimed to characterize the spatial and temporal changes of heavy metals released from industrial areas in public waters based on the PRTR in Korea, and to evaluate the relationships between the effluents of heavy metals and industry types. 

## 2. Materials and Methods

### 2.1. Chemical Data

We obtained a dataset with nine heavy metals (copper: Cu, lead: Pb, manganese: Mn, zinc: Zn, cadmium: Cd, chromium: Cr, tin: Sn, antimony: Sb, and nickel: Ni) from the PRTR system (http://ncis.nier.go.kr/tri/) operated by the Ministry of Environment of Korea from 1999 to 2010. The system provides the monitoring results for 426 chemicals in 15 administrative regions of Korea: Gyeonggi (GG), Gangwon (GW), Chungbuk (CB), Chungnam (CN), Gyeongbuk (GB), Gyeongnam (GN), Jeonbuk (JB), Jeonnam (JN), Seoul (SU), Inchon (IC), Daejoen (DJ), Daegu (DG), Gwangju (GJ), Ulsan (US), and Busan (BS). Those chemicals are divided into two groups according to the annual amounts handled (e.g., group I: more than 1 ton/year and group II: more than 10 ton/year). In addition, to study the relations between the effluents of heavy metals and industry types, we surveyed industrial types based on the PRTR system (http://ncis.nier.go.kr/tri/) and categorized industrial types into nine types (chemical products, metal products, textiles, electronic components, machinery and equipment, food products, non-metallic mineral products, transport equipment, and others) based on its main products (http://ncis.nier.go.kr/tri/).

### 2.2. Modeling Process

A self-organizing map (SOM) was used to characterize spatial and temporal differences of nine heavy metals effluents released from industries to open aquatic systems in Korean on a nationwide scale. The SOM averages the dataset in weight vectors through a learning process while removing noise [23]. The weight vectors in SOM tend to approximate the probability density function of the input vector and can be used for efficient clustering and visualization [24,25]. Visualization of weight vectors displays the contribution of each input variable to clusters on the trained SOM.

The SOM consists of two layers (input and output layers) that are connected by connection intensities (weights). The input layer formed by computation units (neurons) receives input data and then calculates the distance between the weight vector and input vector by Euclidean distance. The output layer consisted of N output neurons on a two-dimensional hexagonal lattice. The number of output neurons was set to 45 (N = 9 ´ 5) map units by the heuristic equation [26]. Two criteria, the quantization error for resolution and the topographic error for topology preservation, were used to evaluate the map quality [25]. These error values were used as an indicator of the accuracy of the mapping in the preserving topology [27]. 

After the learning process, the SOM units were classified based on a hierarchical cluster analysis using Ward’s linkage method with the Euclidean distance measure [28]. In this study, the total annual effluent of nine heavy metals in 15 different regions for the 12 years from 1999 to 2010 was used for the SOM training. The effluent of heavy metals was transformed by natural logarithm to reduce variation after adding one to all data to avoid the problem of logarithm zero being undefined. The functions provided from the SOM toolbox [29] were used in Matlab Ver. 6.1 [30] for training the SOM. Multi-response permutation procedure (MRPP) was conducted to evaluate the significance of the clusters [31]. The MRPP is a nonparametric procedure for testing the hypothesis of no difference between groups defined by the SOM and was conducted using PC-ORD Ver. 4.25 [32].

Kruskal-Wallis (K-W) and Dunn’s multiple comparisons tests were conducted to compare the differences in the effluent of nine heavy metals among clusters defined by the SOM using statistical software Statistica Ver. 7 [33].

## 3. Results

### 3.1. Changes in Released Amount of Materials

**Figure 1:**
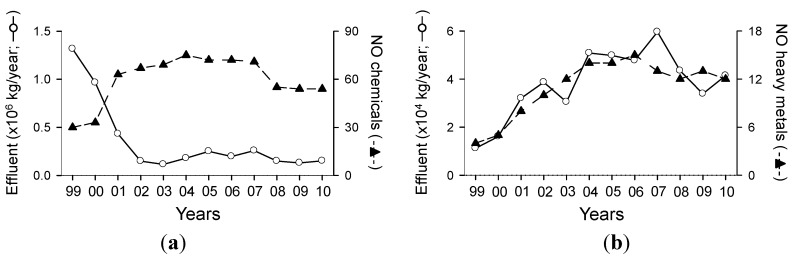
Annual changes of effluent volumes and the number of chemicals released to public waters from 1999 to 2010. (**a**) All chemicals and (**b**) heavy metals.

The number of chemicals reported in PRTR was 30 in 1999, increased to a maximum of 75 in 2004, and decreased to below 55 after 2008 (Figure 1). The number of heavy metals also showed a similar pattern to that for total chemicals. However, the effluents of total chemicals and of heavy metals showed different temporal trends in their volumes. The total volume of chemicals displayed a decreasing trend from 1999 to 2010, whereas that of heavy metals displayed an increasing trend from 1999 (11,183 kg/year) to 2007 (59,633 kg/year).

**Figure 2:**
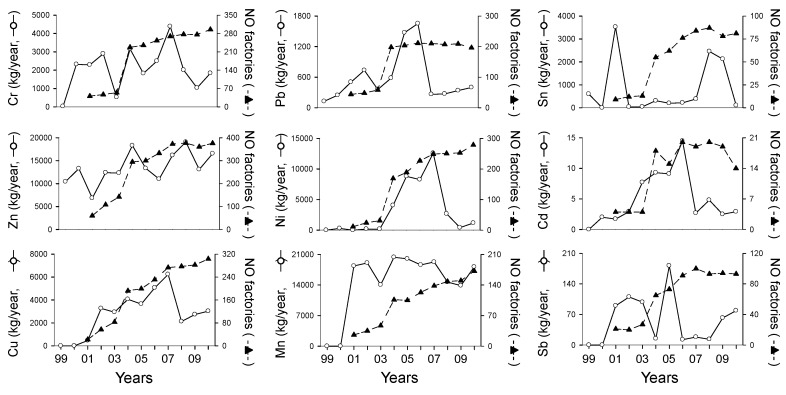
Changes of the number of industrial factories and effluents of nine heavy metals from 1999 to 2010.

**Figure 3:**
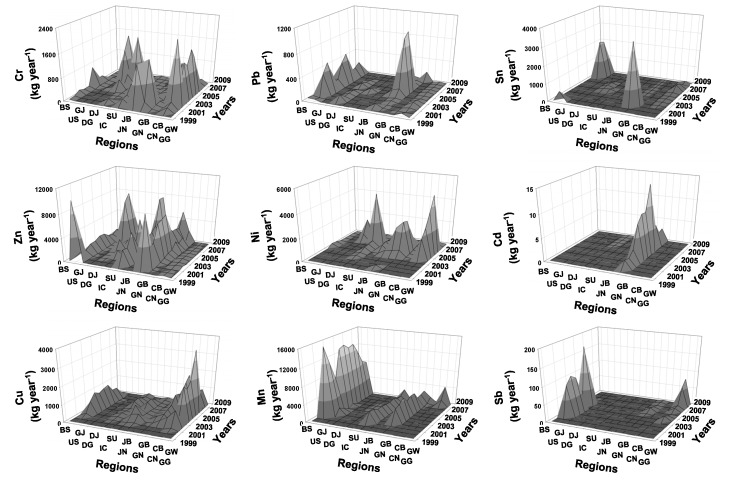
Changes of effluents of nine heavy metals in different regions from 1999 to 2010. GG: Gyeonggi, GW: Gangwon, CB: Chungbuk, CN: Chungnam, GB: Gyeongbuk, GN: Gyeongnam, JB: Jeonbuk, JN: Jeonnam, SU: Seoul, IC: Inchon, DJ: Daejoen, DG: Daegu, GJ: Gwangju, US: Ulsan, and BS: Busan.

The number of industrial factories discharging heavy metals increased dramatically after 2003 for all heavy metals (Figure 2). The effluents of heavy metals were significantly correlated with the number of factories (*r* = 0.31, *p* < 0.01). Meanwhile, different heavy metals displayed different patterns of effluent volumes. Mn was reported in the PRTR after 2001, and it displayed a slight change during the study period, with the exception of 2003, 2008, and 2009. Effluent volumes of Pb and Cd were high in 2006, and those of Ni, Cr, and Cu were at a maximum value in 2007. Mn, Zn, and Sb displayed the highest effluent volumes in Ulsan (Figure 3), whereas other heavy metals showed relatively high values at Gyeonggi (Cr, Ni, and Cu) and Gyeongbuk (Pb, Sn, and Cd).

Nine heavy metals displayed significant relationship with each other in their effluent volumes (Table 1). For example, Mn was highly correlated with Zn (*r* = 0.55, *p* < 0.01) and Sb (*r* = 0.53, *p* < 0.01), but it was not significantly correlated with Cr, Sn, and Ni (*r* = 0.11, *r* = 0.14 and *r* = 0.11, respectively, *P* > 0.05). Sb was positively correlated with Pb (*r* = 0.52, *p* < 0.01) and Cu (*r* = 0.50, *p* < 0.01).

**Table 1:** Spearman rank correlation coefficients between effluents of nine heavy metals.

Heavy Metals	Cr	Pb	Sn	Zn	Ni	Cd	Cu	Mn
Pb	0.18 ^*^							
Sn	0.33 ^**^	0.29 ^**^						
Zn	0.37 ^**^	0.29 ^**^	0.25 ^**^					
Ni	0.36 ^**^	0.01	0.18 ^*^	0.15				
Cd	0.18 ^*^	0.17 ^*^	0.33 ^**^	0.28 ^**^	0.19 ^*^			
Cu	0.35 ^**^	0.22 ^**^	0.25 ^**^	0.33 ^**^	0.33 ^**^	0.21 ^*^		
Mn	0.11	0.17 ^*^	0.14	0.55 ^**^	0.11	0.30 ^**^	0.49 ^**^	
Sb	0.21 ^*^	0.52 ^**^	0.47 ^**^	0.32 ^**^	0.08	0.26 ^**^	0.50 ^**^	0.53 ^**^

*: *p* < 0.05; **: *p* < 0.01.

**Figure 4:**
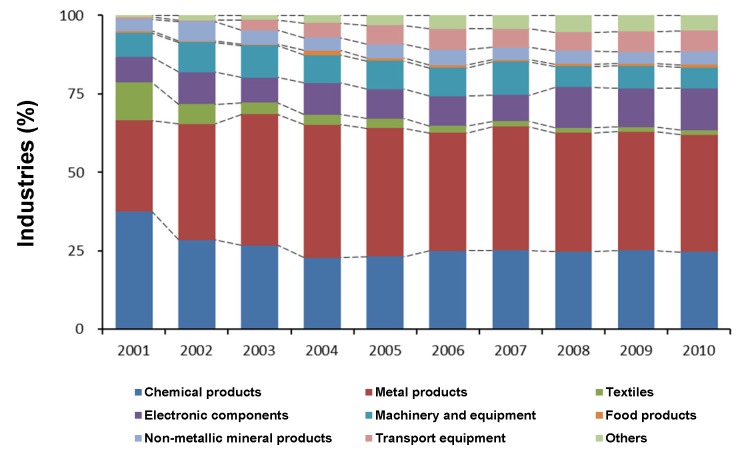
Changes of industrial types from 2001 to 2010.

From 2001 to 2010, the dominant Korean industries were the manufacture of chemical products and the manufacture of metal products (Figure 4). However, their ratios among the total industries discharging heavy metals decreased after 2001 and 2003, as the number of industrial factories was dramatically increased. Meanwhile, the ratios of the manufacture of transport equipment and others were gradually increased.

### 3.2. Differences of Nine Heavy Metals in Space and Time

**Figure 5:**
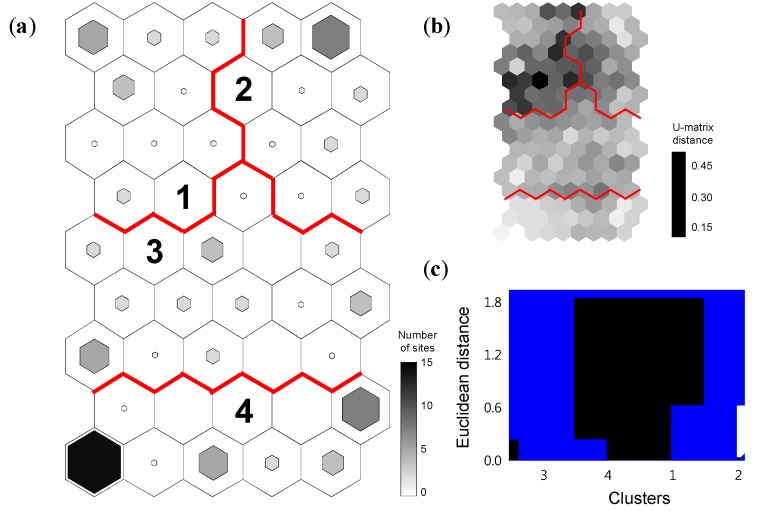
(**a**) Classification of samples based on nine heavy metals released into freshwater ecosystems in Korea from 1999 to 2010 through the training of self-organizing map (SOM), (**b**) U-matrix, and (**c**) dendrogram of the hierarchical cluster analysis with the Ward linkage method using Euclidean distance for the SOM units.

The trained SOM characterized the differences of heavy metal effluents in space and time (Figure 5). The final quantization and topographic errors of the SOM learning were 0.432 and 0.014, respectively, indicating that the SOM was smoothly trained in topology. Based on the dissimilarities in the dendrogram of hierarchical cluster analysis and U-matrix, the SOM output units were classified into four clusters (1-4) that differed significantly (MRPP, *A* = 0.31, *p* < 0.001) (Figure 5). The number of samples in each cluster was visualized as the size of the hexagonal lattice in each SOM unit. The clusters showed differences in the effluent of nine heavy metals among study regions. The relative ratio of samples from Ulsan and Gyeongbuk were higher in cluster 1, whereas the ratio of samples from Gyeonggi and Chungnam were higher in cluster 2. Most of the samples in cluster 3 consisted of samples from Busan, Gwangju, Daejeon, and Gyongnam. Meanwhile, samples in cluster 4 were widely distributed in Korean on a nationwide scale except for Ulsan, Gyeongbuk, Jeonnam, and Gyeonggi.

**Figure 6:**
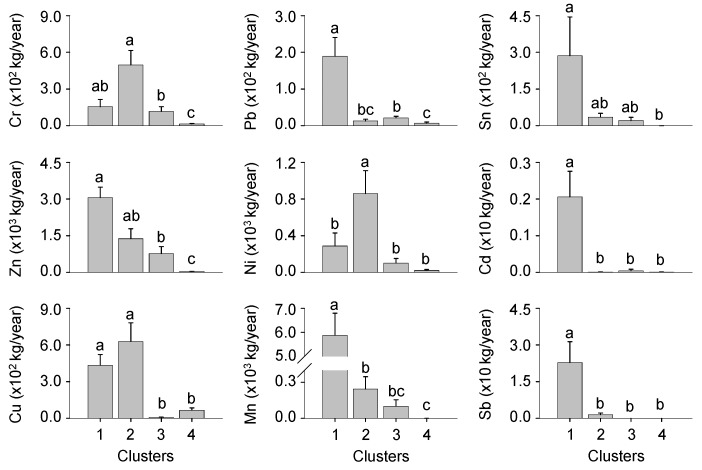
Differences in nine heavy metals among the four clusters defined in the SOM. Different letters indicate significant differences between the clusters based on Dunn’s test after a Kruskal–Wallis test (*p* < 0.05). Error bars indicate standard error.

**Figure 7:**
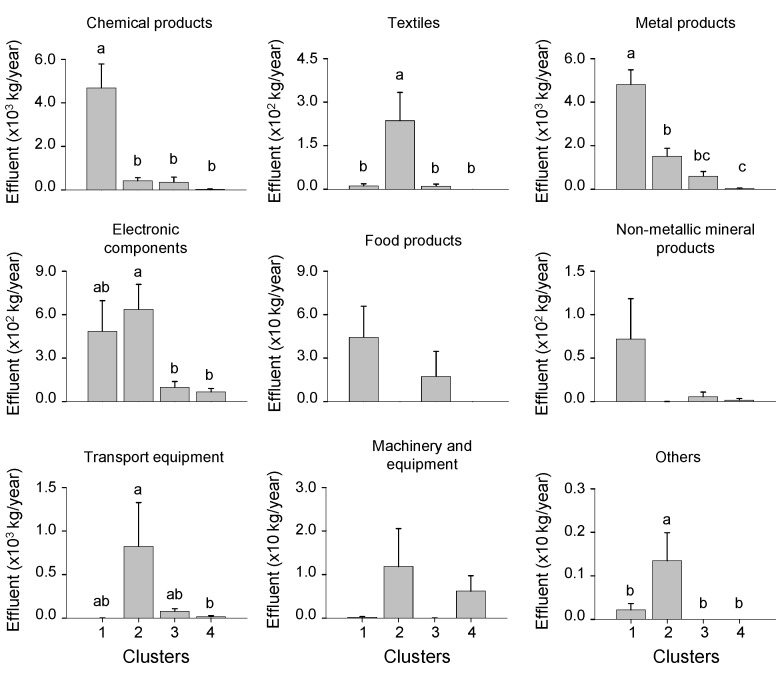
Differences in effluent of heavy metals for each industry types among the four clusters defined by the SOM. Different letters indicate significant differences between the clusters based on Dunn’s test after a Kruskal–Wallis test (*p* < 0.05). Error bars indicate standard error.

The effluent volumes of nine heavy metals were significantly different among clusters (Dunn’s test, *p* < 0.05) (Figure 6). The effluent volumes of Pb, Cd, Mn, and Sb were significantly higher in cluster 1 than in other clusters, and those of Cd and Sb showed no significant differences among three different clusters (clusters 2-4) (Dunn’s test, *p* < 0.05). Meanwhile the effluents of Cr, Ni, and Cu were the highest in cluster 2 and the lowest in cluster 4, with the exception of Cu (Dunn’s test, *p* < 0.05). Similarly, the effluents of heavy metals from manufactures of chemical products and metal products were high in cluster 1, whereas those from manufactures of textiles, electronic components, transport equipment, and others were high in cluster 2 (Figure 7). Clusters 3 and 4 were characterized by small effluents of heavy metals. Meanwhile, effluent volumes from manufactures of food products, non-metallic mineral products, and machinery and equipment were not significantly different among clusters (Dunn’s test, *p* > 0.05). The number of industries also differed between clusters (Table 2). The number of industries of different industrial types was significantly higher in clusters 1 and 2 than in cluster 4 (Dunn’s test, *p* < 0.05).

**Figure 8:**
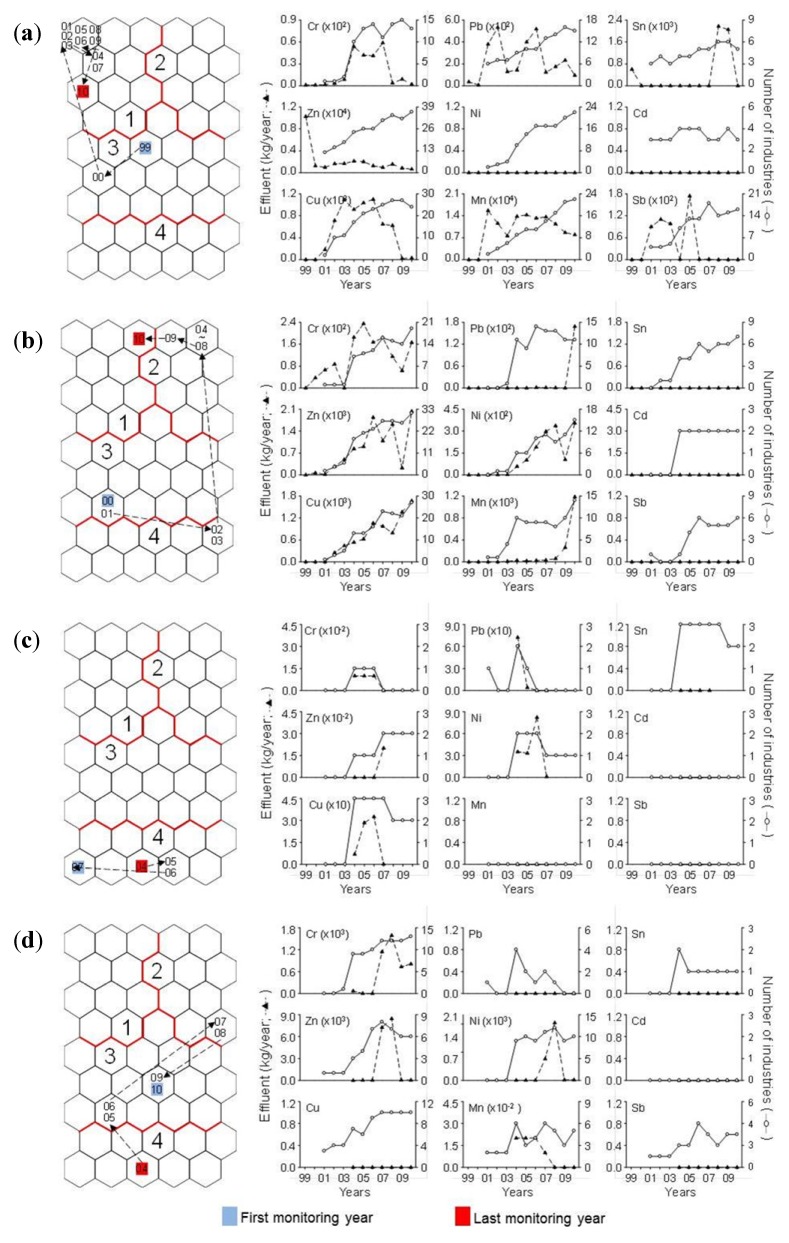
Examples of temporal changes in the effluent patterns of heavy metals in the SOM map and annual changes in the effluents of nine heavy metals at (**a**) Ulsan, (**b**) Chungnam, (**c**) Seoul, and (**d**) Daegu. Two digit numbers in the SOM maps indicate years (for example, 99: 1999, 01: 2001, *etc.*).

The effluents of heavy metals were different in space and time (Figure 8). For instance, the samples from Ulsan in 1999 and 2000, showing the highest effluent volume in heavy metals among 15 different regions, were located in cluster 3, representing relatively low effluent volume in heavy metals, whereas the samples after 2001 were in cluster 1. This was due to an increase in the number of factories releasing heavy metals in Ulsan (Figure 8). The samples from Chungnam shifted from the bottom area of the SOM map (cluster 3: 2000 and 2001, cluster 4: 2002 and 2003) to the upper area of the map (cluster 2: 2004–2009 and cluster 1: 2010). The effluent volumes of heavy metals in Chungnam showed similar pattern with the number of factories in Cu and Ni. Meanwhile, most samples from Daegu were found in cluster 3 with the exception of 2004 (cluster 4), 2007 and 2008 (cluster 2), whereas the samples from Seoul for 2004–2007 were limited to cluster 4. These regions showed relatively low effluent volume in heavy metals with small number of factories.

**Table 2:** Differences in the number of industries (standard error) at different industry types at different clusters defined in SOM. Different letters indicate significant differences between the clusters based on Dunn’s test after a Kruskal–Wallis test (*p* < 0.05).

Industrial Types	SOM Clusters			
	1	2	3	4
Chemical products	28.6 (4.9) ^a^	28.5 (5.7) ^a^	12.7 (2.5) ^b^	5.2 (1.0) ^b^
Metal products	44.7 (7.1) ^a^	48.2 (7.7) ^a^	17.7 (3.7) ^b^	7.9 (2.6) ^b^
Textiles	1.8 (0.6) ^ab^	3.8 (1.0) ^a^	1.3 (0.4) ^ab^	0.2 (0.1) ^b^
Electronic components	16.4 (3.8) ^ab^	12.3 (2.9) ^a^	1.8 (0.4) ^bc^	3.5 (0.6) ^c^
Machinery and equipment	10.1 (1.5) ^a^	7.6 (1.1) ^ab^	5.1 (1.1) ^bc^	1.9 (0.6) ^c^
Food products	1.0 (0.4) ^a^	1.5 (0.4) ^a^	0.2 (0.1) ^ab^	0.0 (0.0) ^b^
Non-metallic mineral products	8.0 (2.0) ^a^	2.8 (0.5) ^a^	1.6 (0.5) ^b^	0.8 (0.2) ^b^
Transport equipment	4.6 (1.0) ^ab^	6.8 (1.2) ^a^	3.3 (0.5) ^ab^	2.2 (0.5) ^b^
Others	7.5 (1.3) ^a^	4.4 (1.0) ^a^	0.6 (0.2) ^b^	0.7 (0.2) ^b^

## 4. Discussion

Large industrial complexes have been built in Korea since the 1960s according to the national policy prioritizing economic growth. Even though the expansion of industrialization has achieved dramatic industrial and economic development, the rapid industrialization has caused severe environmental pollution and industrial disasters. For example, 93 serious industrial accidents were reported in Korea for a 10 year period (1988 to 1997) [34]. These accidents led to an increase of social concerns about both the environmental problems related to the industrialization as well as with instilling a sense of occupational safety. As a result, regulations and laws concerning environmental pollutions have legislated and strengthened, especially focusing on industrialization. In addition, public interest and environmental organizations pressed for the general availability of information related to toxic chemicals [18]. Consequently, monitoring and prevention of heavy metal pollution has received considerable attention due to their toxicity and potential bioaccumulation of heavy metals in many aquatic species and in humans [3,7,35,36]. Wang *et al*. [2] mentioned that the health risks posed by exposure to heavy metals of local inhabitants in Tianjin, China who were threatened by consumption of heavy metals accumulated in both vegetables and fish. Additionally, understanding changes of heavy metal effluent related to industrial type is important for management of heavy metals released into aquatic ecosystems. Seo *et al*. [37] reported that the ecotoxicology effect of industrial effluent differed depending on types of business. In this study, we characterized the spatial and temporal changes of heavy metals released from industrial areas to public waters based PRTR system on a nationwide scale through the SOM analysis, and evaluated the relationships between the effluents of heavy metals and industrial types.

To reflect this social interest and the changes of the social system in response to the OECD council’s request [16], the Ministry of Environment (MOE) in Korea investigates environmental pollutants from various facilities, and the surveyed information has been opened to the public through the PRTR system. The establishment of the PRTR system and the intensification of the government's environmental policy have made the investigation of chemical substances discharged into the environment (air, water, and soil) to be strengthened. These government’s efforts were well reflected in the changes of effluents in PRTR system in this study, showing different temporal changes of different chemicals. Even though the number of discharged materials into the environment largely increased (*i.e.*, 60 in 1999 to 242 chemicals in 2011) [17,38], the number of chemicals released from industrial areas to public waters increased dramatically in 2001 (63 chemicals) and decreased to below 55 chemicals after 2008 (Figure 1). The total volume of chemicals released from industrial areas to public waters was 1,315,884 kg/year in 1999 and decreased to under 300,000 kg/year after 2001 (Figure 1). These differences were caused by the PRTR system settlement and the strengthening of legislation. In fact, 16 chemicals such as sodium hydroxide and arsenic and its compounds were not released into tributaries after 2007 because of strengthened regulations and laws [38]. 

The effluent volumes of heavy metals were high in cluster 1, which mainly include the samples from Ulsan and Gyeongbuk, whereas low in cluster 4, which consist of samples widely distributed in Korea on a nationwide scale except Ulsan, Gyeongbuk, Jeonnam, and Gyeonggi (Figure 6). These differences are due to regional differences in industrial structure and scale of industrial complex. For instance, Ulsan, which mostly is located in cluster 1, is one of the most representative industrial complexes in Korea including heavy industry and automobile industry, whereas Gangwon, which is located in cluster 4, is composed of small local industrial complex. The changes in industrial structure in same region led to changes of the effluents of heavy metals. For instance, the number of factories and the effluents of heavy metals in Seoul were decrease after the mid 2000s (Figure 8), and it was related to changes of industrial structure in Seoul. In fact, Seoul Digital Industrial Complex (Guro Industrial Park), which is representative industrial area in Seoul, is transformed and accelerated to the technology-intensive small and medium enterprises and knowledge-based service firms after the mid 2000s [39]. In addition, the effluents of heavy metals were significantly correlated with the number of factories (*r* = 0.31, *p* < 0.01) (Figure 2). Park *et al*. [40] also reported that the industrial area in Seoul, Daegu, and Gangwon were analyzed to be declining from 2001 to 2008, whereas other regions (Busan, Inchon, Gwangju, Daejoen, Ulsan, Gyeonggi, Chungbuk, Chungnam, Jeonbuk, Jeonnam, Gyeongbuk, and Gyeongnam) showed expansion on their industrial areas. These results agree with our results. The industrial complexes in Seoul and Daegu in clusters 3 and 4 have reduced their factories with relatively low effluent volume in heavy metals, whereas the number of factories in Chungnam and Ulsan showed increased pattern (Figure 8). 

Our results showed clearly the differences of heavy metals effluents at different regions in different years, reflecting the differences of industrial structure and size of industrial complexes as well as the efforts such as strengthening the environmental standards and policies for monitoring aquatic ecosystems to reduce pollution level. The improvement of environmental condition might be promoted by social concerns with increase of economic situation and environmental education in Korea. 

## 5. Conclusions

This study was conducted to characterize spatial and temporal changes of heavy metals released into open water systems based on PRTR system in Korea. The number of industrial factories discharging heavy metals increased dramatically after 2003 for all heavy metals. The heavy metal effluents released into freshwater ecosystems were classified into four clusters through the learning process of the SOM. Cluster 1 was characterized by the relatively higher effluent volumes of heavy metals, whereas cluster 4 had lower effluent volumes. The different patterns of the effluent volumes in heavy metals were closely associated with the differences of industrial types, and the changes of effluents of heavy metals reflected the changes in regulations and laws for aquatic ecosystem management.

## References

[1] Lee Y.H., Stuebing R.B. (1990). Heavy metal contamination in the River Toad, juxtasper (Inger), near a copper mine in East Malaysia. Bull. Environ. Contam. Toxicol..

[2] Wang X., Sato T., Xing B., Tao S. (2005). Health risks of heavy metals to the general public in Tianjin, China via consumption of vegetables and fish. Sci. Total Environ..

[3] Zhou Q., Zhang J., Fu J., Jiang G. (2008). Biomonitoring: An appealing tool for assessment of metal pollution in the aquatic ecosystem. Anal. Chim. Acta.

[4] Sekabira K., Origa H.O., Basamba T.A., Mutumba G., Kakudidi E. (2010). Heavy metal assessment and water quality values in urban stream and rain water. Int. J. Environ. Sci. Tech..

[5] Bae M.-J., Kim J.S., Park Y.-S. (2012). Evaluation of changes in effluent quality from industrial complexes on the Korean nationwide scale using a self-organizing map. Int. J. Environ. Res. Public Health.

[6] Hickey C.H., Clements W.H. (1998). Effects of heavy metals on benthic macroinvertebrate communities in New Zealand streams. Environ. Toxicol. Chem..

[7] Pourang M. (1995). Heavy metal bioaccumulation in different tissues of two fish species with regards to their feeding habitats and trophic levels. Environ. Monit. Assess..

[8] Introduction to Heavy Metal Monitoring. http://pollutantdeposition.defra.gov.uk/heavy_metals.

[9] Al-Juboury A.I. (2009). Natural pollution by some heavy metals in the Tigris River, Northern Iraq. Int. J. Environ. Res..

[10] Jefferies D.J., Freestone P. (1984). Chemical analysis of some coarse fish from a Suffolk River carried out as part of the preparation for the first release of captive-bred otters. J. Otter. Trust.

[11] Heath A.G. (1987). Water Pollution and Fish Physiology.

[12] Toxics Release Inventory (TRI) Program. http://www.epa.gov/tri/.

[13] Lynn F.M., Kartez J.D. (1994). Environmental democracy in action: The toxics release inventory. Environ. Manage..

[14] Arora S., Cason T.N. (1999). Do community characteristics influence environmental outcomes? Evidence from the Toxics Release Inventory. South. Econ. J..

[15] Kerret D., Gray G.M. (2007). What do we learn from emissions reporting? Analytical considerations and comparison of pollutant release and transfer registers in the United States, Canada, England, and Australia. Risk Anal..

[16] Organisation for Economic Co-operation and Development (1996). Pollutant Release and Transfer Registers (PRTRs) Guidance Manual for Governments.

[17] Korean Ministry of Environment (2003). The Survey of the Effluent in Chemical Substance Released to Environment Report 2001.

[18] Park H., Chah S., Choi E., Kim H., Yi J. (2002). Releases and transfers from petroleum and chemical manufacturing industries in Korea. Atmos. Environ..

[19] Kim J.H., Kwak B.K., Park H.S., Kim N.G., Choi K., Yi J. (2010). A GIS-based national emission inventory of major VOCs and risk assessment modeling-Part I-methodology and spatial pattern of emissions. Korean J. Chem. Eng..

[20] Babich H., Stotzky G. (1985). Heavy metal toxicity to microbe-mediated ecologic processes: A review and potential application to regulatory policies. Environ. Res..

[21] Burton G.A. (1991). Assessing the toxicity of freshwater sediments. Environ. Toxicol. Chem..

[22] Qu X., Wu N., Tang T., Cai Q., Park Y.-S. (2010). Effects of heavy metals on benthic macroinvertebrate communities in high mountain streams. Ann. Limnol. Int. J. Lim..

[23] Vesanto J., Himberg J., Siponen M., Simula O. Enhancing SOM Based Data Visualization. Proceeding of the 5th International Conference on Soft Computing and Information/Intelligent Systems (IIZUKA’98).

[24] Kohonen T. (2001). Self-Organizing Maps.

[25] Park Y.-S., Céréghino R., Compin A., Lek S. (2003). Applications of artificial neural networks for patterning and predicting aquatic insect species richness in running waters. Ecol. Model..

[26] Vesanto J., Alhoniemi R. (2000). Clustering of the self-organizing map. IEEE Trans. Neural Networ..

[27] Céréghino R., Park Y.-S. (2009). Review of the Self-Organizing Map (SOM) approach in water resources: Commentary. Environ. Modell. Softw..

[28] Legendre P., Legendre L. (1998). Numerical Ecology.

[29] SOM Toolbox: Laboratory of Information and Computer Science, Helsinki University of Technology. http://www.cis.hut.fi/projects/somtoolbox.

[30] Mathworks. www.mathworks.com.

[31] Mielke E.W., Berry K.J., Johnson E.S. (1976). Multi-response permutation procedures for a priori classifications. Commun. Stat. Theor. M..

[32] McCune B., Mefford M.J. (1999). PC-ORD: Multivariate Analysis of Ecological Data.

[33] StatSoft: Making the World More Productive. www.statsoft.com.

[34] Kang S.J. (1999). Trends in major industrial accidents in Korea. J. Loss Prevent. Proc..

[35] Gupta B.N., Mathur A.K. (1983). Toxicity of heavy metals. Indian J. Med. Sci..

[36] Blevins R.D. (1985). Metal concentrations in muscle of fish from aquatic systems in east Tennessee, USA. Water Air Soil Pollut..

[37] Seo J.W., Lee S.K., Lee H.J., Yoon H.G., Lee S.O. Ecotoxicological Evaluation of Complex Industrial Effluents with Whole Effluent Toxicity (WET) in Korea. Proceeding of the Joint Conference of Korean Society on Water Environment and Korean Society of Water and Wastewater.

[38] Korean Ministry of Environment (2013). The Survey of the Effluent in Chemical Substance Released to Environment Report 2011.

[39] Koo Y. (2012). An analysis of cluster life cycle in the dynamics evolution of the Seoul Digital Industrial Complex in Korea. J. Korean Assoc. Reg. Geogr..

[40] Park B.H., In B.C., Kim T.Y. (2009). Analysis on the decline of industrial area in Korea. J. Korean Reg. Sci. Assoc..

